# Protein recovery from inclusion bodies of *Escherichia coli* using mild solubilization process

**DOI:** 10.1186/s12934-015-0222-8

**Published:** 2015-03-25

**Authors:** Anupam Singh, Vaibhav Upadhyay, Arun Kumar Upadhyay, Surinder Mohan Singh, Amulya Kumar Panda

**Affiliations:** Product Development Cell, National Institute of Immunology, Aruna Asaf Ali Marg, New Delhi, 110067 India

**Keywords:** Inclusion body, Recombinant protein, Solubilization, Refolding

## Abstract

Formation of inclusion bodies in bacterial hosts poses a major challenge for large scale recovery of bioactive proteins. The process of obtaining bioactive protein from inclusion bodies is labor intensive and the yields of recombinant protein are often low. Here we review the developments in the field that are targeted at improving the yield, as well as quality of the recombinant protein by optimizing the individual steps of the process, especially solubilization of the inclusion bodies and refolding of the solubilized protein. Mild solubilization methods have been discussed which are based on the understanding of the fact that protein molecules in inclusion body aggregates have native-like structure. These methods solubilize the inclusion body aggregates while preserving the native-like protein structure. Subsequent protein refolding and purification results in high recovery of bioactive protein. Other parameters which influence the overall recovery of bioactive protein from inclusion bodies have also been discussed. A schematic model describing the utility of mild solubilization methods for high throughput recovery of bioactive protein has also been presented.

## Introduction

High level expression of recombinant protein in *Escherichia coli* often results in aggregation of the expressed protein molecules into inclusion bodies [1-3]. Use of high temperature during protein expression, high inducer concentration and expression under strong promoter systems often results in expression of the desired protein at a high translational rate. This exhausts bacterial protein quality control system and the partially folded and misfolded protein molecules aggregate to form inclusion bodies [4]. Reduced environment of bacterial cytosol, lack of eukaryotic chaperones and post-translational machinery also contribute to the formation of inclusion bodies [5]. Although in recent years bacterial inclusion bodies have been reported to provide many biotechnological applications [6], the emphasis of this current review is to elaborate upon the recovery of recombinant proteins from inclusion bodies particularly using mild solubilization processes.

Formation of inclusion bodies imposes a great hurdle in production and purification of recombinant proteins using *E. coli* as host [7-9]. Inclusion bodies need extensive processing involving isolation from cell, solubilization, refolding and purification to produce the bioactive proteins. In spite of new developments in understanding of the structural details of protein in inclusion bodies, most of the times, solubilization and refolding is carried out in empirical ways leading to poor recovery of functional protein. Solubilization of inclusion body proteins and their refolding can be fine tuned in accordance with the new information about the structural and functional characteristics of inclusion body proteins. In *E. coli* due to specificity of protein aggregation, inclusion bodies mostly consist of the target recombinant protein of interest. If a convenient and efficient way of recovering properly folded protein molecules from inclusion body aggregates can be developed, it will reduce the need of extensive chromatographic purification steps. Inclusion bodies have been shown to have protein molecules in native-like conformation with some inclusion bodies having significant biological activity. Use of high concentration of chaotropes like urea and guanidine hydrochloride (GdnHCl) results in complete denaturation of these existing secondary structures and often leads to aggregation of protein molecules during refolding process.

Recovery and refolding of protein from inclusion bodies using mild solubilization strategies have been reported to be high in comparison to that achieved while solubilizing with high concentration of chaotropes. These mild solubilization agents retain the existing secondary structures of proteins to some extent; inhibit protein aggregation during refolding leading to improved recovery of bioactive proteins. The objective of the present review is to update the recent findings on structural features of proteins in inclusion bodies and new developments in mild solubilization processes for high-throughput recovery of bioactive protein from inclusion bodies of *E. coli*.

### Factors affecting formation of inclusion bodies in *E. coli*

Multiple factors contribute towards the formation of protein aggregates as inclusion bodies*.* In *E. coli,* inclusion bodies accumulate intracellularly as refractile particles with a typical size range of 0.2 to 1.5 μm [10]. Under electron microscope, they appear to be dense, refractile particles with smooth or rough surface morphology [5]. In general, inclusion bodies are spherical but can take cylindrical to ovoid shape to fit the bacterial cell [11]. In *E. coli* they are mostly found to be localized at one or both the poles of bacterial cells [12]. It has been recently reported that localization of inclusion body at the poles of bacterial cell is driven by macromolecular crowding in bacterial cytosol [13]. During cell division only one daughter cell receives the inclusion body and formation of inclusion body in the other cell starts *de novo* [12]. Generally inclusion bodies are accumulated in cytosol, but proteins with secretary signals have also been reported to form aggregates in periplasmic space [14,15].

Expression of recombinant proteins involves multiple synthetic pathways and is regulated at transcriptional and translational levels. Usually, when the level of expression of protein goes beyond 2% of the total cellular proteins, it leads to inclusion body formation [16]. Factors which favor high rate of recombinant protein expression increase the chances of the expressed protein to get aggregated into inclusion bodies. High copy number of target gene, strong promoter system and high inducer concentration favor the formation of inclusion bodies. Too high a copy number costs the cells a high metabolic burden while a low copy number puts forth a risk of plasmid loss in subsequent generations. The promoter strength of the vectors also plays an important role in regulating the expression levels of the proteins. When bacterial cells having a strong promoter system are induced to produce recombinant proteins, they experience an elevated level of metabolic burden with increased chances of expressed recombinant protein to aggregate into inclusion bodies [17,18]. Reduced environment of bacterial cytosol also causes protein aggregation [19]. Formation of inclusion bodies also depends upon amino acid sequence of protein with highly hydrophobic proteins having more chances to aggregate into inclusion bodies [16]. Presence of bacterial chaperones has been widely reported in inclusion bodies [4,20]. It has been shown that absence of cytosolic chaperone, ClpB increases insoluble expression of aggregation prone recombinant proteins [20]. Protein aggregation is a regulated phenomenon in most type of cells [21]. In *E. coli,* these aggregates in the form of inclusion bodies are deposited at poles only [12]. This polar localization result in partition of inclusion bodies in an asymmetric way between the daughter cells [22]. This helps in enhanced cell growth rate of the daughter cells devoid of aggregates and make the bacteria better suited for further division.

### Structural features of proteins in inclusion bodies

The first detailed mechanistic studies on formation of inclusion bodies were conducted by King and colleagues [23]. *In vitro* folding of phage P22 tailspike protein was compared with *in vivo* folding pathway. This lead to the development of a model describing partitioning of polypeptides into folding and aggregation pathways from partially folded intermediate [23]. It is widely accepted that protein aggregation is a highly specific process and protein molecules can only aggregate with other molecules of same or highly related proteins. Inclusion body aggregates are also highly specific in nature as recombinant protein constitutes major fraction of these aggregates. Kopito and co-workers in 2001, employing two different aggregation prone proteins labeled with different fluorescent protein tags, showed using fluorescence resonance energy transfer (FRET) that these proteins do not co-aggregate in eukaryotic cell [24]. Specificity of protein aggregation in bacterial inclusion bodies was further confirmed by expressing amyloid beta with two different fluorescent tags [Blue Fluorescent Protein (BFP) and Green Fluorescent Protein (GFP)], followed by FRET analysis [25]. It was observed that same type of protein molecules co-aggregate in cell whereas different proteins like VP1 do not co-aggregate with amyloid beta [25]. These results showed that protein aggregation in inclusion bodies is a highly specific phenomenon.

Inclusion bodies are highly dynamic in nature and protein molecules participating in inclusion body formation can reversibly disaggregate and fold into their native form [26,27]. There exists a dynamic equilibrium between protein molecules aggregated in inclusion bodies and their soluble counterpart. Thus, a continuous process of construction and deconstruction of inclusion bodies takes place in which protein molecules in aggregated and soluble forms can freely exchange their localization [26].

Classically, inclusion bodies were considered to be amorphous type of aggregates, devoid of any structural regularity. In last decade, a number of reports provided evidences in support of amyloid nature of inclusion bodies [28], though recently it was shown that there can be inclusion bodies which are not amyloid in nature [29]. Inclusion body aggregates have been shown to bind to amyloid specific dyes such as Thioflavin T and Congo Red [28]. Fourier transform infrared (FTIR) spectroscopic analysis reveals that inclusion bodies are rich in beta content and give signals corresponding to cross-beta structure, a characteristic feature of amyloid aggregation [30]. Presence of cross-beta structure in these aggregates is also evident from the X-Ray diffraction pattern given by inclusion bodies which has striking similarity to that generated by amyloid fibrils [31]. Amyloid aggregation is a nucleation based phenomenon and aggregates of a protein can act as seeds for aggregation of similar proteins. This also holds true for *E. coli* inclusion bodies. It has been reported that like amyloid aggregates, inclusion bodies can seed *in vitro* aggregation of similar proteins [25]. Inclusion bodies incubated with soluble protein have been shown to be associated with long fibrillar structures having morphological similarity with amyloid fibrils [25]. It has also been reported using H/D exchange NMR that amyloid formation in inclusion body aggregates is a residue specific phenomenon [31]. Even a point mutation can inhibit the process of inclusion body formation [15,32]. There are several online servers which predict regions which have high propensity to form amyloids. Hamodrakas and co-workers have recently applied such a tool to predict polypeptide regions which are responsible for the formation of inclusion bodies [33].

Bacterial inclusion bodies have been reported to have native-like secondary structures [27]. Proteins like Interleukin-1β, β-lactamase, Human granulocyte-colony stimulating factor (GCSF) form inclusion bodies having native-like secondary structure [27,34,35]. Initial studies reporting presence of native-like secondary structures primarily used FTIR spectroscopy [34]. Solid state NMR spectroscopy has also been used to demonstrate the presence of native-like structure in inclusion bodies [36]. Moreover, a large number of inclusion bodies have been shown to have considerable biological activity [37,38]. Although the first report suggesting presence of activity in inclusion bodies came in 1989 [39], it is in the last decade that active inclusion bodies have grabbed the attention due to their potential applications in various processes [40-45]. Inclusion bodies of β-lactamase, β-galactosidase, and GCSF have been shown to have considerable biological activity [27]. Inclusion body aggregates which are biologically active are known as non-classical inclusion bodies [46]. Most of the non-classical inclusion bodies are characterized by a loose arrangement of protein molecules and thus can be solubilized even at low concentration of denaturants [47]. The proportion of active molecules inside inclusion bodies depends upon physical conditions used during protein expression. Quality of inclusion bodies can be modulated by changing expression temperature. Although formation of active inclusion bodies at high temperature has also been reported [41], it is generally considered that low expression temperature favors the formation of non-classical inclusion bodies [46,48].

Formation of active inclusion bodies can be induced by linking a protein to aggregation prone peptides by suitable linkers. Wu *et al.* demonstrated that linking a self-assembling ionic peptide ELK16 (LELELKLK)_2_ to proteins can lead to formation of inclusion bodies with significant biological activity [49]. Surfactant like peptides have also been used to form biologically active inclusion bodies [50]. Attachment of full length green fluorescent protein as an inducer of aggregation for the formation of active inclusion bodies and importance of the linker peptides has also been reported [51].

Inclusion body aggregates are of highly dynamic nature. Besides the recombinant protein, inclusion bodies also contain components from bacterial membrane, other host proteins and RNA [52]. As mentioned earlier, inclusion bodies contain amyloid-like proto-fibrils or fibrils. Riek and coworkers using NMR spectroscopy have shown that a fraction of proteins in inclusion bodies have H/D exchange rates different than those of amyloid content [31]. This indicated that there exists a structural heterogeneity in inclusion bodies. This heterogeneity can be due to contaminating host proteins. There is also a possibility that recombinant protein molecules in unfolded, partially folded or even native structures can co-exist in inclusion bodies and in part are responsible for the structural heterogeneity of protein aggregates in inclusion bodies. This also provides an explanation for the presence of activity in some inclusion bodies. The current theoretical model of inclusion bodies assumes that they are primarily made up of amyloid-like proto-aggregates. Recombinant protein molecules in other conformations, including the native state, are trapped in the network of amyloid-like proto-fibrils or fibrils [11]. The proportions of amyloid-like component and other structural forms of recombinant protein depend upon a number of factors including the physical conditions used during expression [53]. All these new information strongly suggest that inclusion body proteins have considerable amount of native-like structures.

### Recovery of bioactive proteins from inclusion bodies

As protein molecules are in an aggregated state in inclusion bodies, it is a challenging task to solubilize inclusion bodies and refold the solubilized proteins into bioactive form [8,9,54]. The conventional strategy to purify proteins from inclusion bodies consists of four major steps: isolation of purified inclusion bodies, solubilization of inclusion bodies, refolding of solubilized proteins and purification of refolded proteins by various chromatographic techniques [55]. Solubilization of inclusion bodies and refolding of solubilized protein molecules into native conformation are the most crucial steps in the recovery of bioactive protein from inclusion bodies.

#### Isolation of purified inclusion bodies from *E. coli* cells

Inclusion bodies are highly specific aggregates and are mostly composed of recombinant protein of interest. It is thus necessary to isolate and purify inclusion body aggregates into homogeneity before solubilization and refolding. Purification of proteins from inclusion bodies reduces the need of tags and multiple chromatographic steps. As discussed above, expression conditions affect the quality of inclusion bodies. Low expression temperatures can help in formation of soft, non-classical inclusion bodies which can be solubilized using non-denaturing solvents [46,48]. Regulating the pH during expression has been reported to affect inclusion body quality [56]. Methods used for the isolation of inclusion bodies from bacterial cells include mechanical cell rupture using sonication or French press and chemical cell disruption methods which make use of cell lysis agents like lysozyme. The choice of cell disruption method has been reported to make a great impact on quality of inclusion bodies [47]. Cell disruption by chemical methods is supposed to be better in comparison to the mechanical methods like sonication or homogenization. The latter affect the quality of inclusion bodies and lead to aggregation of protein molecules which were initially a part of soluble fraction [47]. Use of suitable combination of mechanical and chemical cell disruption techniques have also been reported [57]. Inclusion bodies, due to their high density in comparison to other cellular components can be isolated from the whole cell lysate by centrifugation [57]. Cross-flow membrane microfiltration has also been used for isolating inclusion bodies from host cellular proteins [58]. Isolated inclusion bodies contain several impurities like host proteins, RNA and host membrane fragments [52]. They are further purified using several washing steps. Washing with low concentrations of detergents like deoxycholic acid and Triton X-100 [54] not only helps in achieving highly purified inclusion bodies, but also helps in removing membrane fragments. Non-classical inclusion bodies have been reported to be very sensitive to pH at which they are purified, as use of high pH can lead to solubilization of protein molecules during purification [53,57]. Sucrose density gradient ultracentrifugation has also been used for purification of inclusion bodies [57]. Pure inclusion bodies help in reducing the interference of contaminants during refolding process and reduce the requirement of extensive purification steps.

#### Solubilization of inclusion body proteins

Traditionally, inclusion bodies are solubilized using high concentration of denaturants and chaotropes like urea and guanidine hydrochloride (GdnHCl) [8,59]. For proteins containing multiple cysteine residues, β-mercaptoethanol or dithiothreitol are added in these solubilization agents to reduce incorrect disulfide bonds. Solubilization of inclusion bodies using high concentration of chaotropes results in complete disruption of protein structure. This, in some cases, leads to aggregation of protein molecules during refolding process [60]. As inclusion body aggregates have been shown to have native-like secondary structures and can have activity, it is advantageous to use a “mild” solubilization process which does not completely unfold these native-like protein structures [61].

As mentioned earlier, inclusion bodies are dynamic in nature and there exists an equilibrium between folded and aggregated protein molecules. This fact can be utilized for solubilizing inclusion bodies in non-denaturing buffers without assistance of any solubilization agent. Inclusion bodies of N-acetyl-d-glucosamine 2-epimerase have been solubilized using Tris–HCl buffer at pH 7. The solubilized protein was reported to be active [62]. Biologically active protein molecules can be extracted from non-classical inclusion bodies using mild solubilization conditions. This process preserve the native-like protein structures present in inclusion bodies and thus bypasses the refolding steps. Low concentrations of organic solvents like 5% n-propanol and DMSO and detergents like 0.2% N-lauroyl sarcosine has been used as mild solubilization agents for solubilization of non-classical inclusion bodies [46]. Low concentration of urea in many cases has also been used to solubilize inclusion body aggregates [46,63]. These solubilization agents result into extraction of active recombinant protein without any requirement of refolding step. The major drawback of these methods is their limitation to work on classical inclusion bodies.

**Figure 1:**
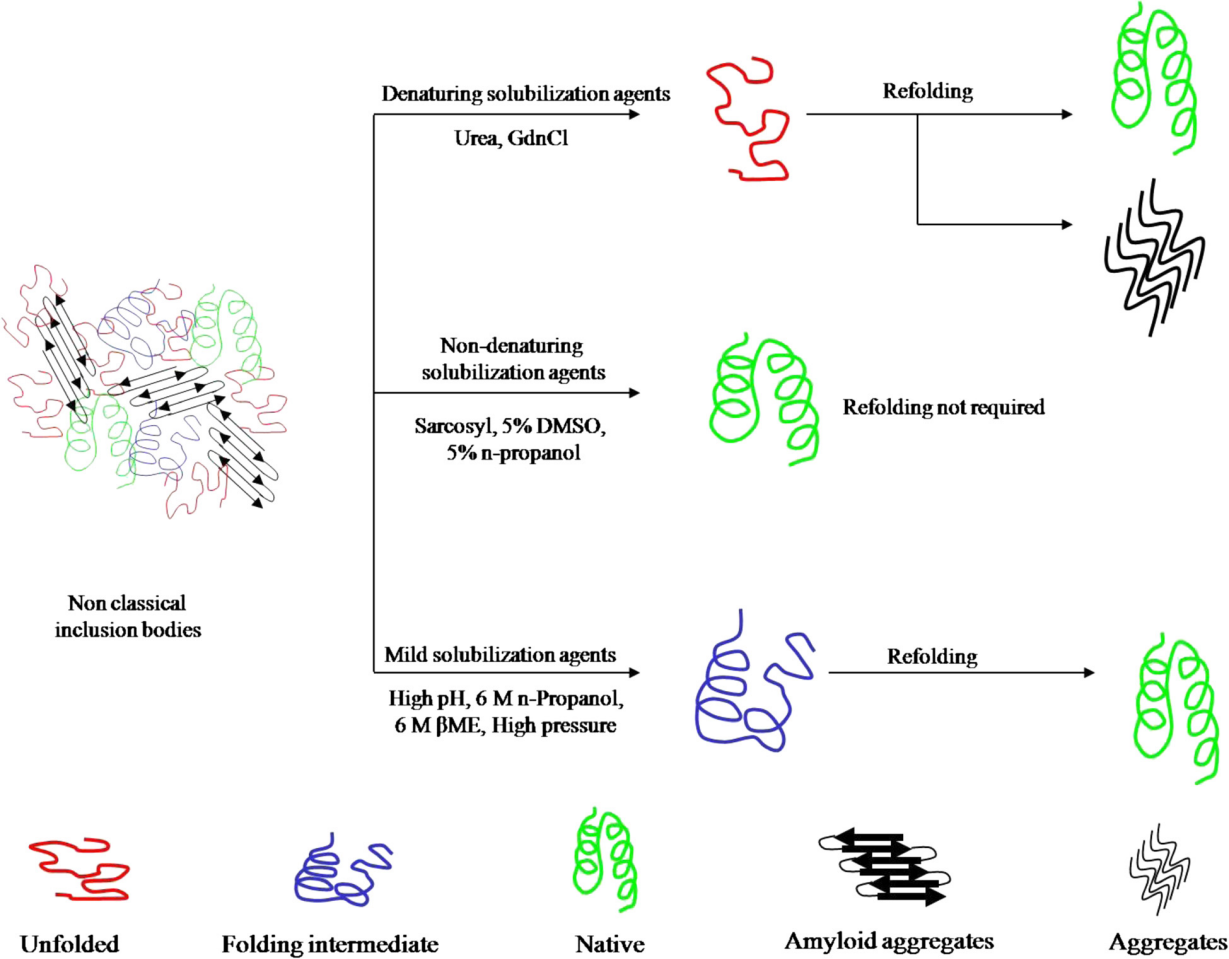
**Model showing different solubilization methods used for recovery of protein from inclusion bodies.**

For efficient solubilization of inclusion bodies, several mild solubilization methods have been developed which keep the protein molecules in partially folded state during solubilization. With these methods, refolding of solubilized protein molecules starts from a partially folded form, inhibiting the molecules from going into aggregation pathway during refolding. The schematic diagram representing these mild solubilization methods is given in Figure 1. Mild solubilization processes using alkaline pH [19,61], high pressure [64], detergents [65,66], organic solvents [60] and low concentration of chaotropes [46,63] have been used for recovery of bioactive proteins from inclusion bodies. Table 1 provides the summary of these methods. In most of the cases, improvements in inclusion body isolation and use of modern refolding methods in combination with mild solubilization improved the overall recovery of bioactive proteins.

**Table 1:** **Mild solubilization methods used for recovery of proteins from inclusion bodies**

**Solubilization method**	**Remarks**	**Reference**
Tris-Cl buffer, low concentration of DMSO, n-propanol and Sarcosyl	Non-denaturing solubilization methods for non-classical inclusion bodies. No need of refolding	[46,62]
Low concentration of urea	Mild solubilization, low concentration of urea doesn’t completely denature solubilized protein molecules	[46,63]
Organic solvent based solubilization	High solubilization efficiency, inhibition of aggregation during refolding	[60]
High hydrostatic pressure	High solubilization efficiency with simultaneous refolding of solubilized protein	[64,68]
High pH buffers	High pH in combination with low concentration of urea. Mild, efficient solubilization	[19,61]
Detergents	Mild solubilization	[65,66]

High hydrostatic pressure also helps in solubilization of inclusion body aggregates [64]. High pressure (2–4 kbar) disrupts the intermolecular interactions and disaggregates inclusion bodies. Removal of applied high pressure leads to refolding of protein molecules [64,67]. Solubilization of inclusion bodies applying 2.4 kbar pressures at −9°C and subsequent refolding at 0.4 kbar and 20°C has also been shown to improve refolding of recombinant endostatin [68].

Use of buffers with extreme pH has also been reported as a mild solubilization method. High pH (>12) buffer in combination with 2 M urea has been used successfully for solubilization of inclusion bodies [19,61]. High pH buffer has been described to improve refolding yield by retaining native-like secondary structures in solubilized state [19]. Solubilization with low concentrations of urea [63], detergents like N-Lauroylsarcosine and Lauroyl-L-glutamate have also been reported to improve the yield of bioactive protein from inclusion bodies [65,66,69].

Use of organic solvents like β-mercaptoethanol (βME) [70] and n-propanol in combination with low concentration of urea has been reported as a novel solubilization strategy for improved recovery of proteins [60]. Both the solvents have been used to solubilize human growth hormone inclusion bodies. In case of human growth hormone, use of n-propanol based buffer enhances refolding yield over the conventionally used urea or GdnHCl based buffers by preserving native-like secondary structure [60]. Organic solvents, mainly alcohols have long been known to be interacting with proteins and affect their secondary and tertiary structure. Many alcohols have also been shown to have a stabilizing effect on the protein secondary structure and are known to induce helicity even in non structured peptides [71,72]. Solubilization of inclusion body aggregates using organic solvent thus provides a viable alternative to urea/GdnHCl based solubilization. A variety of proteins have been reported to be solubilized by a mixture of n-propanol and 2 M urea [60]. This opens up new possibilities for the improved recovery of proteins from bacterial inclusion bodies using organic solvent based solubilization.

No single method of solubilization works for every protein and thus, screening for solubilization agents for optimized solubilization has to be carried out. For a fast and convenient screening of solubilization agents, Hahn and co-workers have reported a turbidity based high-throughput assay to screen a large number of solubilization agents [73]. Mild solubilization of inclusion body aggregates by understating the dominant force that cause protein aggregation is thus the key for successful development of high throughput protein refolding process from inclusion bodies of *E. coli.*

#### Refolding of solubilized protein

Solubilized inclusion body proteins are refolded by removal of solubilization agent. Dilution of the solubilized protein in refolding buffer [59] and dialysis of the solubilized protein in presence of refolding buffer [74] are the most common methods used to recover functionally active proteins. These methods suffer from disadvantages like high buffer requirement particularly for large scale operation and low refolding yields due to protein aggregation. Low protein concentration and reduced intermolecular interactions during refolding are a prerequisite for inhibiting protein aggregation and increasing the refolding yield. To achieve this, a modified method for dilution known as pulsatile dilution has been developed, which significantly reduces the buffer requirement and improves the refolding yield of proteins [54].

To improve the quality of the refolded protein and scale up the process for industrial application, refolding in packed chromatographic columns has been developed. Different chromatographic methods have been used for refolding of solubilized proteins. Size exclusion [75-79], ion exchange [80-83] and affinity chromatography [84-86] are the most extensively used methods, while hydrophobic interaction chromatography [87,88] has also been used in some of the cases. On column refolding, as it is called, has several advantages over conventional methods of dilution and dialysis. Use of size exclusion chromatography for refolding results in separation of the folded form from the aggregated and misfolded forms during elution with the refolding buffer. In other chromatographic methods, refolding process occurs after immobilization of the protein on the solid support that leads to spatial separation of the refolding units and decreased intermolecular interactions. Thus, refolding processes in chromatographic beds can be carried out at high protein concentrations. Also, the refolding process is coupled with denaturant removal and purification of the target protein. This decreases the number of steps involved in the purification procedure and automates the process [89,90]. There are reports of using chaperonins immobilized on the chromatographic media with a view of mimicking *in vivo* folding [91-95]. Although, an increase in refolding yield is observed, the industrial application is limited due to high process cost. Continuous developments in the field have improved the process and the recent advances in the methods are aimed at increasing the refolding yield [96] and optimization of the refolding conditions to improve the quality of the protein [97].

A new method of refolding has been recently described that involves the use of microfluidic chips [98]. In microfluidic chips the denaturant concentration is maintained by controlling diffusion through laminar flow in microchannels and has been used for refolding of difficult to fold protein like citrate synthase. Another method of protein refolding using urease enzyme has also been described that involves gradual removal of urea from the solubilized protein solution mediated by urease enzyme catalyzed reaction [99]. A major advantage of such a system is that efficient protein refolding can be achieved in low volume without the use of refolding buffers that can significantly decrease the cost of production. Different methods used for refolding of solubilized inclusion body proteins are summarized in Table 2.

**Table 2:** **Methods used for refolding of solubilized inclusion body proteins**

**Refolding methods**	**Remarks**	**Reference**
Dilution		
Flash dilution	Simplest method	[59]
Pulsatile dilution	low requirement of buffer and improved refolding yield	[54]
Dialysis		
One step dialysis	May be successful only for those proteins that are soluble in intermediate states	[74]
Step wise dialysis	Useful for multidomain or disulphide bond containing proteins	[74]
On column refolding		
Size exclusion chromatography	Separation of folded form from intermediates	[75-79]
Anion exchange chromatography	More advantageous for crude samples	[80-83]
Affinity chromatography	Limited to cases where the Tag doesn’t interfere with folding	[84-86]
Hydrophobic interaction chromatography	May substitute for the requirement of additives during refolding	[87,88]
Chromatography in presence of chaperones	Reduces aggregation by mimicking *in vivo* scenario	[91-95]
Micro fluidic chips	May be useful for difficult to fold proteins	[98]
Urease mediated refolding	No requirement of refolding buffer	[99]

The success of the method employed for refolding depends in part on the composition of refolding buffer. The most common problem during refolding is the aggregation of the target protein and most of the refolding strategies involve the use of certain additives in refolding buffer to inhibit aggregation. Apart from aggregation inhibitors, certain folding enhancers are also used to improve the yield. Common additives used in refolding buffer include chaotropic agents like urea [19] or guanidine hydrochloride [74] in low concentrations, amino acids like glycine [100], arginine [101,102] and proline [103,104], polyhydric alcohols and sugars like polyethylene glycol [105-108], glycerol [104,109], sorbitol [110] and sucrose [104]. Non detergent zwitterions like sulfo-betaines, substituted pyridines and pyrroles and acid substituted amino cyclohexanes have also been used as additives during refolding [111-114]. These additives are summarized in Table 3.

**Table 3:** **List of additives commonly used to promote refolding of solubilized proteins**

**Common additives used in refolding buffer**	**References**
Chaotropes	
Urea	[19]
Guanidine hydrochloride	[74]
Amino acids	
Glycine	[100]
Arginine	[101,102]
Proline	[103,104]
Sugars and polyhydric alcohols	
Sucrose	[104]
Polyethylene glycol	[105-108]
Sorbitol	[110]
Glycerol	[104,109]
Others	
Sulfobetaines	[111-114]
Substituted pyridines and pyrolles	
Acid substituted aminocyclohexanes	

It is known that refolding of some proteins *in vivo* require the presence of N-terminal pro-peptide between the signal peptide and the mature part of polypeptide [115]. Some strategies of refolding *in vitro* exploit the addition of the pro-peptide in the refolding buffer to increase the refolding yield [116-118]. Similarly, other strategies inspired from protein folding *in vivo* exploit chaperones in refolding of solubilized proteins. Chaperones are natural protein folding aiding machinery. They act by sequential capture and release of the refolding intermediates and preventing them from interacting with each other. The use of chaperones and other folding catalysts like peptidylprolyl cis-trans isomerase or protein-disulfide isomerase, that accelerate the rate limiting steps on the folding pathway, have been shown to improve the refolding yield [91-95]. Detergent micelles acting as artificial chaperon systems have been used in improving the yield of refolding process. Here the protein folding intermediates interact with detergents to form mixed protein-detergent micelles and are protected from intermolecular interactions. The refolding of the intermediate form is initiated by addition of cyclodextrins that strip the detergents from the protein-detergent complex by forming more stable detergent cyclodextrin-complex [119,120]. Recently, a strategy to purify and refold proteins was described based on separation of the proteins using affinity ligands bound to smart polymers [121-124]. These polymers precipitate on providing specific stimulus like temperature, pH or change in ionic strength, thus precipitating the protein of interest. The bound proteins are then recovered from the polymers by reversing the precipitating conditions. There is no universal buffer for optimal protein refolding and the composition of the refolding buffer has to be screened for each case individually [9,125-126].

## Conclusions

Advanced structural techniques have significantly enhanced our understanding of protein structure in inclusion body aggregates. Inclusion bodies are now considered to have conformational heterogeneity, with amyloid structures building a network in which protein molecules with other conformations, including the native ones, are trapped. Mild solubilization methods have been developed in order to preserve these native-like structures and provide a viable alternative to the conventional use of high concentration of chaotropes. Efficient recovery of folded protein also reduces the burden of extensive chromatographic steps. Combined with improved refolding methods, mild solubilization results in high-throughput recovery of bioactive proteins from inclusion bodies. The process of protein recovery from inclusion bodies involves four steps: 1. Purification of inclusion bodies to homogeneity (careful lysis of cells, purify inclusion bodies by detergent washing/ultracentrifugation), 2. Solubilization of inclusion bodies using mild solubilization agent (alkaline pH/hydrostatic pressure/organic solvent based buffers/2-3 M urea/detergents), 3. Refolding of the solubilized proteins (refolding at high protein concentration by pulsatile renaturation/on column refolding/urease mediated refolding) with optimal refolding buffer and 4. Purification of the refolded protein. These steps can be optimized to recover high amount of bioactive protein from the inclusion bodies of *E. coli*.

## Abbreviations

BFP: Blue fluorescent protein
DMSO: Dimethyl sulphoxide
FTIR: Fourier Transform Infrared
FRET: Fluorescence resonance energy transfer
GCSF: Granulocyte-colony stimulating factor
GdnHCl: Guanidine hydrochloride
GFP: Green fluorescent protein
H/D: Hydrogen/Deuterium
NMR: Nuclear Magnetic Resonance
RNA: Ribose nucleic acid
βME: β-mercaptoethanol
